# Hospital admissions for acute drug poisoning in adults and children: a 7-year retrospective analysis of hospital discharges at a tertiary center

**DOI:** 10.3389/ftox.2025.1672470

**Published:** 2025-10-01

**Authors:** Daniel Wang, Lina Camacho-Arteaga, Rosario Muñoz Gallarín, Immaculada Danés, Antònia Agustí Escasany

**Affiliations:** ^1^ Department of Pharmacology, Therapeutics and Toxicology, Universitat Autònoma de Barcelona, Barcelona, Spain; ^2^ Department of Clinical Pharmacology, Hospital Universitari Vall d’Hebron, Barcelona, Spain; ^3^ Department of Clinical Pharmacology, Hospital del Mar, Hospital del Mar Research Institute, Barcelona, Spain; ^4^ Department of Medical Documentation, Hospital Universitari Vall d’Hebron, Barcelona, Spain

**Keywords:** drug-related side effects and adverse reactions, overdose, poisoning, hospital records, public health surveillance, self-injurious behavior, adult drug poisoning, pediatric drug poisoning

## Abstract

**Background:**

Drug poisoning is a growing public health concern, affecting both adult and pediatric populations. The COVID-19 pandemic has further influenced the incidence and patterns of these episodes. This study describes the clinical and epidemiological characteristics of drug poisoning episodes in adult and pediatric patients treated at a tertiary hospital in Spain between 2018 and 2024.

**Materials and methods:**

This retrospective, observational, single-center study used data from the Spanish Minimum Basic Data Set of Hospital Discharges (CMBD-AH). All hospitalizations coded with a diagnosis of drug poisoning (ICD-10-ES: T36–T50) were included. Variables analyzed included demographics, type of admission, drug class involved, intentionality, length of stay, ICU admission and duration, and clinical outcomes.

**Results:**

A total of 2,989 episodes with at least one drug poisoning code were identified in 2,481 patients (85.7% adults; 14.3% pediatric). The median age was 55 years in adults and 14 years in pediatric patients. Females predominated in both groups. Self-poisoning was the most frequent intentionality (52.4% in adults; 54.7% in pediatric patients), while accidental poisonings were more common in pediatric patients under 12 and adults over 60. Benzodiazepines were the most frequently involved drug-class across all age groups; in pediatric self-poisoning, paracetamol was most commonly implicated. ICU admission was required in 9.6% of pediatric and 9.2% of adult episodes. Mortality was reported in 3.3% of adult and 0.5% of pediatric episodes. Additionally, 12.5% of patients experienced recurrent episodes. A significant post-COVID increase in poisoning episodes was observed (p < 0.001).

**Conclusion:**

Although drug poisoning represented only 1.7% of all hospital discharges, it posed a substantial burden due to its frequency, recurrence, and ICU requirements. The CMBD-AH is a valuable tool for characterizing drug-related hospitalizations across age groups. Strengthened toxicovigilance, targeted prevention strategies, and early mental health interventions are essential to reduce its impact on healthcare systems.

## 1 Introduction

Drug poisoning (DP) represents a significant clinical and public health concern due to its prevalence, diverse clinical manifestations, and potential for severe outcomes. In Spain, as well as in other regions such as the United States and Ethiopia, DP accounts for approximately 40% of poisoning consultations (Gummin et al., 2017; Getie and Belayneh, 2020). It results from exposure to pharmacological substances that cause biological harm through biochemical mechanisms, typically over a short period (Hu et al., 2010; Resiere et al., 2020).

Suspected DP cases in Spain are primarily managed in hospital emergency departments, primary care centers, and prehospital emergency services (Baeza et al., 2020). The recurrent nature of these episodes and their higher prevalence among young adults underscore their importance as a public health issue with substantial socioeconomic, cultural, and psychological implications (Supervía A et al., 2021; Aguilón-Leiva et al., 2022).

Both adult and pediatric populations are affected by DP, although clinical profiles differ markedly. Among adults, episodes are frequently related to intentional self-poisoning, often in the context of mental health disorders. In contrast, pediatric poisonings are more commonly accidental, usually due to unsupervised access to medications or dosing errors (Agarwal et al., 2020; Aguilón-Leiva et al., 2022). Nevertheless, there is growing concern about the increase in intentional self-poisoning among adolescents, typically involving over-the-counter painkillers or psychotropic medications (Fernández Lázaro JC et al., 2022).

Clinical implications vary widely depending on the drug involved, the intentionality, the drug dose, and patient-related factors. While most cases are mild and managed on an outpatient basis, a substantial proportion requires hospital admission, and the most severe cases require intensive care due to life-threatening toxicity (Burillo Putze G et al., 2003).

Despite its clinical relevance, comprehensive data on DP in Spain remain scarce. Most toxicology consultations to the National Institute of Toxicology and Forensic Sciences originate from non-health professionals, leading to underrepresentation of hospital-based cases (Burillo Putze G et al., 2008). Early research was mostly single-center or limited to primary care settings. To address this gap, multicenter initiatives such as SEMESTOX and HISPATOX were launched, integrating data from emergency departments across Spain. These studies found that DP accounted for up to 50% of poisoning cases treated in adults, particularly those involving benzodiazepines in intentional episodes (Rubio González et al., 2016; Baeza et al., 2020). Although pediatric data are more limited, available studies report similar proportions, with paracetamol (acetaminophen) and central nervous system depressants being the most frequently involved drugs (Azkunaga B et al., 2012; Brock et al., 2024).

In parallel, the Toxicovigilance System coordinated by the Spanish Foundation of Clinical Toxicology (FETOC, by its Spanish acronym) has contributed to the systematic monitoring of poisoning cases in emergency and intensive care units since 1999 (Ferrer et al., 2000). However, Spain still lacks a unified national registry comparable to the National Poison Data System (NPDS) in the United States or the National Poisons Information Service (NPIS) in the United Kingdom, hindering national data consolidation and representativeness (Gummin et al., 2017). Accurate epidemiological characterization of DP remains essential to inform prevention strategies, optimize clinical management, and guide health policy.

Notably, the incidence of DP increased progressively until early 2020. The declaration of a nationwide state of emergency in Spain on 14 March 2020, led to a temporary decline in emergency department visits related to DP, particularly during lockdown phases. However, this reduction was short-lived. Subsequent reopening periods were marked by a rebound in poisoning cases—especially those with suicidal intent. This trend has been attributed to increased psychological distress, social isolation, and limited access to mental health services during the pandemic (Caballero-Bermejo et al., 2022; Fernández Lázaro JC et al., 2022).

Considering this evolving context, the present study aims to describe the epidemiological and clinical characteristics of patients diagnosed with DP at a major tertiary hospital in Spain, across both adult and pediatric populations, and covering both pre-pandemic and post-pandemic periods, using hospital discharge data from the Spanish Minimum Basic Data Set (CMBD-AH).

## 2 Materials and methods

### 2.1 Study design and population

This retrospective, observational, single-center study was conducted at Vall d’Hebron University Hospital (VHUH), a tertiary referral center in Catalonia, Spain, and covered the period from 1 January 2018, to 31 December 2024. The study population included all patients admitted during this period with at least one diagnosis of DP.

Patients were classified as pediatric (<18 years) or adult (≥18 years) based on their age at the time of admission.

### 2.2 Data sources

Data were extracted from the Spanish Minimum Basic Data Set of Hospital Discharges (CMBD-AH), a comprehensive, validated administrative database that systematically collects information on all hospitalizations and major surgical procedures across Spain (Lopez-de-Andres et al., 2021). According to CMBD-AH criteria, hospital admissions are classified as either urgent or scheduled.

In scheduled admissions, the reason for hospitalization was unrelated to DP, which instead occurred during the hospital course. In urgent admissions, the poisoning could be the main cause of admission or could arise at any point during the hospital stay in patients admitted through the emergency department. The CMBD-AH database also classifies as hospital admissions those emergency visits lasting more than 16 h.

The CMBD-AH dataset includes patient demographics, hospitalization details, diagnoses, and procedures. Diagnoses were coded according to ICD-10-ES, the Spanish edition of the International Classification of Diseases, 10th Revision. Each episode contains one primary diagnosis and up to 14 secondary ICD-10-ES codes, capturing both pre-existing conditions and complications arising during hospitalization.

### 2.3 Data collection and variables

Episodes were identified from the CMBD-AH of VHUH using diagnostic codes T36-T50, corresponding to drug-related poisonings (see Supplementary Table S1).

Information on the following variables was collected: number of patients and episodes with T36–T50 codes (excluding adverse drug reactions without poisoning), demographic characteristics (age, sex), type of admission (urgent or scheduled), therapeutic group involved (based on ICD-10-ES classification), intentionality of poisoning (self-poisoning, accidental, or undetermined), length of hospital stay (in days), annual number of poisoning episodes, admissions to the intensive care unit (ICU) and their duration (in days), and patient outcomes (death, discharge home, transfer to intermediate care, or voluntary discharge).

Recurrent episodes were defined as distinct poisoning events occurring in the same patient during separate hospital admissions. Notably, the CMBD-AH does not distinguish between multiple distinct DP events occurring within the same hospital admission; it only captures the presence of one or more poisoning-related diagnostic codes.

### 2.4 Statistical analysis and data management

Given the observational nature of the study, primarily descriptive analyses were performed. Categorical variables were summarized using frequencies and percentages, while continuous variables were expressed as medians and interquartile ranges (IQRs). A Mann–Whitney U test, a non-parametric test suitable for skewed distribution, was used to compare the length of ICU stay between adult and pediatric patients. Additionally, a two-sample Z-test for equality of proportions was applied to compare the frequency of DP episodes relative to total hospital admissions between the pre- and post-COVID-19 periods, overall and separately for adult and pediatric hospital discharges.

The Z statistic was calculated as Z = (p1 -p2)/√ [p (1 – p) (1/n1 + 1/n2)], where p1 and p2 are the sample proportions, n1 and n2 their respective sample sizes, and p = (x1 + x2)/(n1 + n2) the pooled proportion. All tests were two-sided, and the alpha level significance was set at 0.05. All analyses were conducted using R software (version 4.4.3 or later) and Microsoft Excel.

### 2.5 Ethical approval

This study was approved by the Clinical Research Ethics Committee of Vall d'Hebron University Hospital, Barcelona, under the reference code EOM(AG)033/2023(6146). It was conducted in accordance with the principles of the Declaration of Helsinki and adhered to Good Clinical Practice guidelines.

## 3 Results

During the 7-year study period, a total of 2,989 episodes of DP were recorded, corresponding to 2,481 unique patients. As each hospital admission was analyzed as a separate episode, some patients contributed more than one episode to the dataset. Overall, DP episodes represented 1.7% of total hospital discharges. Most episodes (n = 2,893; 96.8%) were classified as urgent admissions. The overall median age was 50 years (IQR: 27–71), and 58.4% of patients were female (n = 1,449). Recurrent DP episodes were identified in 309 patients (12.5%), with a total of 817 recurrent episodes (27.3%). General demographic and clinical characteristics are summarized in tables 1 and 2.

**TABLE 1:** Demographic characteristics of patients with acute drug poisoning.

	<18 years old N patient = 355	≥18 years old N patient = 2,126	Total N patient = 2,481
Sex, N (%)			
Male	123 (34.7)	909 (42.8)	1,032 (41.6)
Female	232 (65.3)	1,217 (57.2)	1,449 (58.4)
Age (Median, [IQR])	14 [4–15]	55 [39–74]	50 [27–71]
Distribution of unique patients by age group, N (%)			
<1 year	19 (5.4)	—	19 (0.8)
1–2 years	40 (11.3)	—	40 (1.6)
3–6 years	49 (13.8)	—	49 (2.0)
7–11 years	23 (6.5)	—	23 (0.9)
12–17 years	224 (63.0)	—	224 (9.0)
18–29 years	—	324 (15.2)	324 (13.1)
30–39 years	—	224 (10.5)	224 (9.0)
40–49 years	—	321 (15.1)	321 (13.0)
50–59 years	—	358 (16.8)	358 (14.4)
60–69 years	—	247 (11.6)	247 (10.0)
70–79 years	—	279 (13.1)	279 (11.2)
≥80 years	—	373 (17.7)	373 (15.0)
Patients with ≥ 2-episode, N patient (%)			
2 episodes	25 (75.7)	182 (66.0)	207 (67.0)
3 episodes	5 (15.2)	52 (18.9)	57 (18.4)
4 episodes	3 (9.1)	23 (8.3)	26 (8.4)
5 episodes	—	8 (2.9)	8 (2.5)
6 episodes	—	4 (1.4)	4 (1.3)
7 episodes	—	2 (0.7)	2 (0.7)
9 episodes	—	1 (0.4)	1 (0.3)
10 episodes	—	2 (0.7)	2 (0.7)
11 episodes	—	2 (0.7)	2 (0.7)
Total	33 (100)	276 (100)	309 (100)

**TABLE 2:** Clinical characteristics of acute drug poisoning episodes.

	<18 years old N episodes = 395	≥18 years old N episodes = 2,594	Total N episodes = 2,989
Episodes, N (%)			
Self-poisoning	216 (54.7)	1,359 (52.4)	1,575 (52.7)
Accidental poisoning	132 (33.4)	845 (32.6)	977 (32.7)
Undetermined poisoning	47 (11.9)	390 (15.0)	437 (14.6)
Days of hospitalization (Median, [IQR])	1 [1–6]	2 [1–5]	1 [1–5]
ICU admission, N (%)	38	240	278
ICU length of stay (Median, [IQR])	5 [2–13]	3 [2–7]	3 [2–7]
Hospital discharge destination, N (%)			
Home	314 (79.5)	1,733 (66.8)	2,047 (68.5)
Transfer to an intermediate care facility	77 (19.5)	735 (28.3)	812 (27.2)
Treatment not completed	2 (0.5)	40 (1.5)	42 (1.4)
Death	2 (0.5)	86 (3.3)	88 (2.9)

**FIGURE 1:**
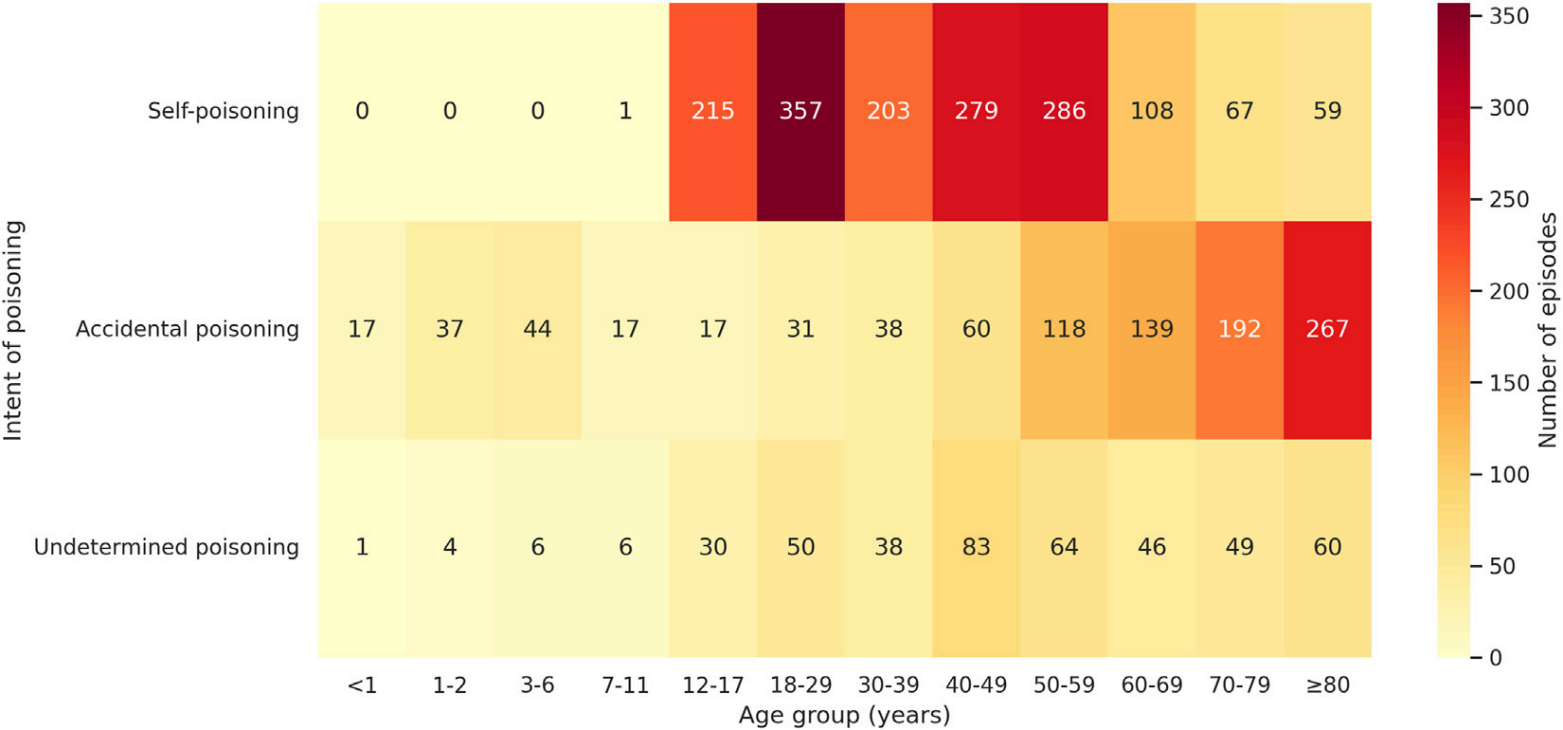
Heatmap of drug poisoning episodes by age group and intentionality. Age groups are expressed in years. The color scale represents the number of episodes in each category.

Among adult patients (n = 2,126; 85.7%) the median age was 55 years (IQR: 39–74), and 1,217 were female (57.2%). In pediatric patients (n = 355; 14.3%), the median age was 14 years (IQR: 4–15), while a higher percentage than in adults were female (n = 232; 65.3%). In both populations, most episodes were urgent admissions (n = 2,529; 97.5% in adults, and n = 364; 92.2% in pediatric patients), while scheduled episodes accounted for 65 (2.5%) and 31 (7.8%) episodes in adults and pediatric patients, respectively (see Table 2). Recurrent episodes were identified in 276 adults (13.0%) and in 33 children (9.3%). The most frequent type of exposure was self-poisoning, both in adults and in pediatric patients (n = 1,360; 52.4% in adults and n = 216; 54.7% in pediatric population), followed by accidental (n = 844; 32.5% in adults and n = 132; 33.4% in pediatric population), and undetermined (n = 390; 15.0% in adults, and n = 47; 11.9% in pediatric patients) exposures (Figure 1).

Regarding clinical departments, the most frequent discharge units overall were psychiatry (n = 1,037; 34.7%) and emergency departments (adult and pediatric combined, n = 992; 33.2%). In adult patients, most episodes were discharged from psychiatry (n = 1005; 38.7%) and adult emergencies (n = 788; 30.4%). In pediatric cases, the leading departments were pediatric emergencies (n = 142; 35.9%) and general pediatrics (n = 84; 21.3%) (Supplementary Table S2).

The overall median hospital stay was 1 day (IQR: 1–5), with a median of 2 days (IQR: 1–5) in adults and 1 day (IQR: 1–6) in pediatric patients. A total of 278 episodes (9.3%) required ICU admission: 240 in adults (9.2%) and 38 in pediatric patients (9.6%). The median ICU stay was significantly longer in pediatric patients (5 days, IQR: 2–13) than in adults (3 days, IQR: 2–7; p = 0.036).

**FIGURE 2:**
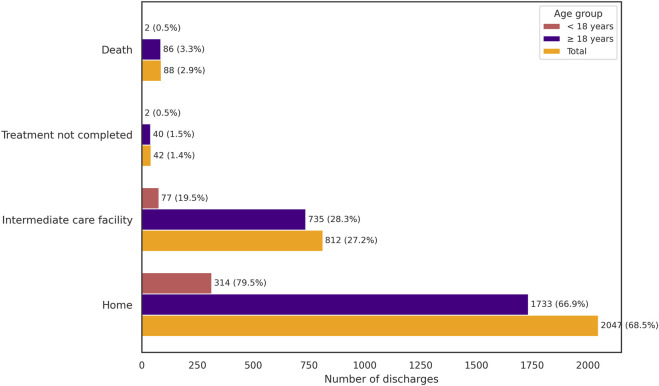
Discharge destinations in drug poisoning episodes, stratified by age group (<18, ≥18 years) and total.

Regarding clinical outcomes, most episodes overall resulted in discharge home (n = 2,047; 68.5%), followed by transfer to an intermediate care facility (n = 812; 27.2%), treatment not completed (n = 42; 1.4%), or death (n = 88; 2.9%). Among adults, 1,733 episodes (66.8%) ended in discharge home, 735 (28.3%) in transfer to intermediate care, 40 (1.5%) in incomplete treatment, and 86 (3.3%) resulted in death. Most adult deaths were associated with accidental poisoning (n = 64), followed by undetermined (n = 17) and self-poisoning (n = 7). Twenty-nine of the deceased adults had been admitted to ICU. In pediatric patients, 314 episodes (79.5%) resulted in discharge home, 77 (19.5%) in transfer to intermediate care, 2 (0.5%) in incomplete treatment, and 2 (0.5%) died—one associated with antineoplastic and immunosuppressive agents, and the other with medications and biological products (Supplementary Table S5). Importantly, the presence of a DP diagnosis does not necessarily imply causality in death or ICU admission (Table 2; Figure 2).

Across all age groups and intentionality, benzodiazepines were the most frequently involved drugs (n = 1,146) followed by paracetamol (n = 215) and antipsychotics/neuroleptics (n = 218) (Tables 3 and 4). Among adults, benzodiazepines were most often implicated in both self-poisoning (n = 763) and accidental episodes (n = 144). In pediatric patients, paracetamol was the leading drug in self-poisoning (n = 79), while benzodiazepines were most frequent in accidental (n = 30) and undetermined (n = 11) episodes. Drug combinations were identified in 319 episodes (11.7%), most of which involved two substances (n = 221; 7.4%), commonly benzodiazepines co-used with steroidal anti-inflammatory drugs, antidepressants, or paracetamol (Supplementary Table S6).

**TABLE 3:** Most common drugs coded involved by intentionality.

Self-poisoning	N (%)	Accidental	N (%)	Undetermined	N (%)
Benzodiazepines	828 (43.4)	Benzodiazepines	174 (17.6)	Benzodiazepines	144 (31.2)
Paracetamol	194 (10.2)	Opioids	155 (15.7)	Medications and Biological Products	84 (18.2)
Antipsychotics and Neuroleptics	170 (8.9)	Cardiotonic Glycosides	92 (9.3)	Opioids	42 (9.1)
Medications and Biological Products	138 (7.2)	Antipsychotics and Neuroleptics	66 (6.7)	Methadone	22 (4.8)
Selective Serotonin Reuptake Inhibitors	97 (5.1)	Medications and Biological Products	58 (5.9)	Narcotics	22 (4.8)
Antidepressants	90 (4.7)	Anticoagulants	57 (5.8)	Antineoplastics and Immunosuppressants	17 (3.7)
Propionic Acid Derivatives	70 (3.7)	Antineoplastics and Immunosuppressants	50 (5.1)	Antiepileptics and Hypnotics	14 (3.0)
Antiepileptics and Hypnotics	66 (3.5)	Narcotics	50 (5.1)	Antipsychotics and Neuroleptics	12 (2.6)
Insulin and Hypoglycemics	28 (1.5)	Insulin and Hypoglycemics	36 (3.6)	Paracetamol	11 (2.4)
Selective serotonin and norepinephrine reuptake inhibitors	20 (1.0)	Antiepileptics and Hypnotics	32 (3.2)	Propionic Acid Derivatives	10 (2.2)
Total codes	1,908 (100)	Total codes	988 (100)	Total codes	462 (100)

The total number of codes may exceed the number of episodes, as a single episode can include more than one recorded poisoning code.

**TABLE 4:** Most commonly coded acute poisonings by age group and intentionality.

Intent	<18 years old N	Intent	≥18 years old N
Self-poisoning			
Paracetamol	79	Benzodiazepines	763
Benzodiazepines	65	Antipsychotics and neuroleptics	160
Propionic acid derivative	40	Medications and biological product	127
Selective serotonin reuptake inhibitors	21	Paracetamol	115
Medications and biological product	11	Antidepressants	84
Accidental poisoning			
Benzodiazepines	30	Opioids	153
Antineoplastics and immunosuppressants	14	Benzodiazepines	144
Paracetamol	11	Cardiotonic glycosides	90
Antipsychotics and neuroleptics	9	Antipsychotics and neuroleptics	57
Antiarrhythmics	6	Anticoagulants	56
Undetermined poisoning			
Benzodiazepines	11	Benzodiazepines	133
Paracetamol	5	Medications and biological products	80
Antifungal	4	Opioids	40
Antineoplastics and immunosuppressants	4	Methadone	22
Antiviral	3	Narcotics	20

The total number of codes may exceed the number of episodes, as a single episode can include more than one recorded poisoning code.

**FIGURE 3:**
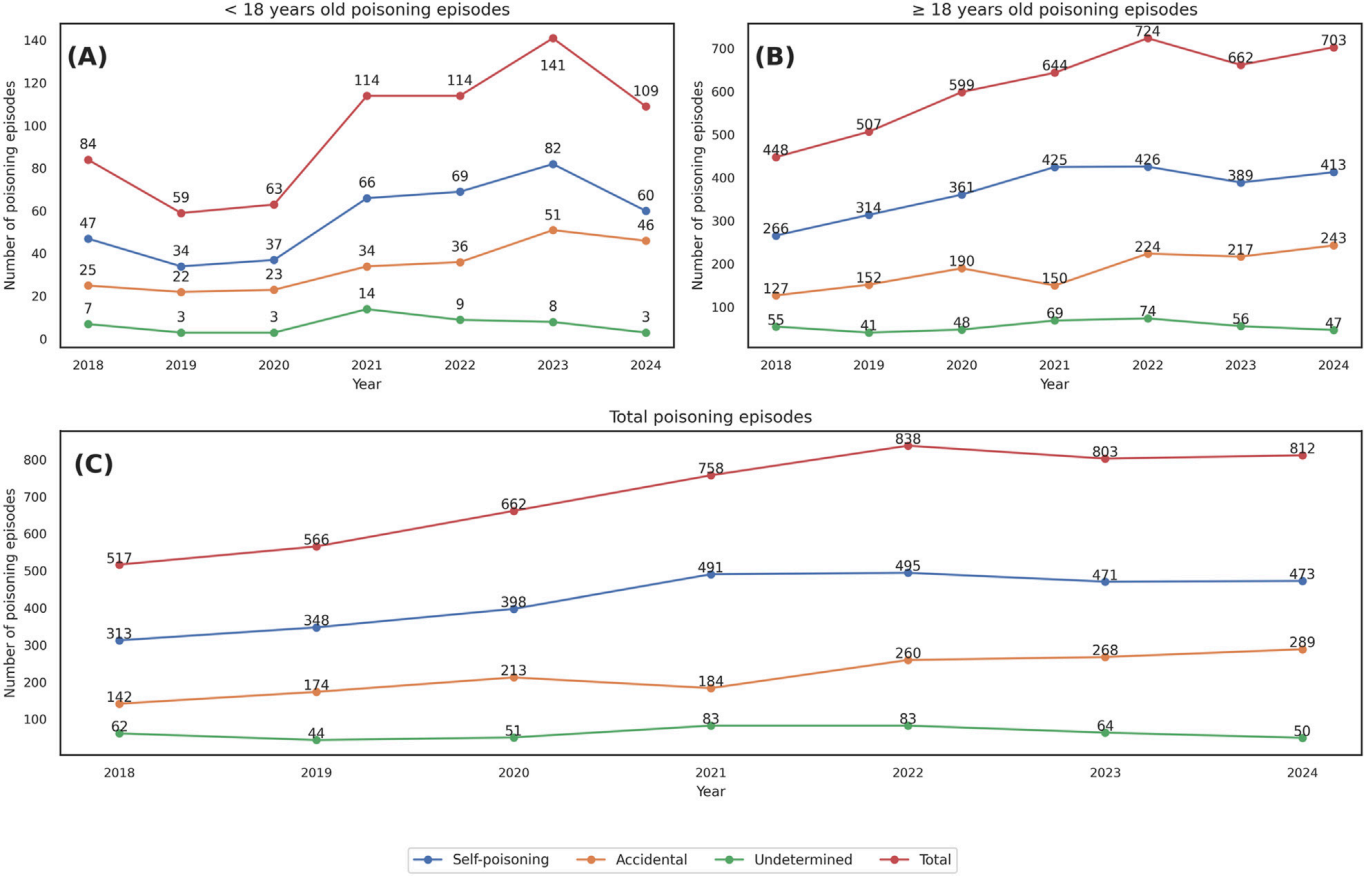
Annual trend of drug poisoning episodes between 2018 and 2024, stratified by age group and intentionality **(A)** Patients aged <18 years. **(B)** Patients aged ≥18 years. **(C)** Total number of poisoning episodes (all ages).

The annual proportion of DP admissions relative to total hospital discharges ranged from 1.03% in 2018 to 2.01% in 2024 (Figure 3). A progressive increase was observed from 2020 onwards, peaking at 2.10% in 2021. A two-sample z-test for equality of proportions confirmed a statistically significant increase in DP frequency in the post-COVID period (2020–2024) compared to the pre-COVID period (2018–2019), both overall and by age group. In the adult population, the proportion rose from 1.11% to 1.93% (z = 11.91, p = 1.08 × 10^−32^); in pediatric patients, from 1.11% to 2.22% (z = 5.76, p = 8.62 × 10^−9^); and in the total sample, from 1.11% to 1.97% (z = 13.18, p = 1.20 × 10^−39^). All differences were statistically significant.

## 4 Discussion

This study describes 2,989 hospital admissions for DP at a large Spanish tertiary hospital over a 7-year period, encompassing both adult and pediatric populations. The median age was 55 years in adults and 14 years in children, with a predominance of female patients in both groups. Recurrent episodes occurred in 12.5% of patients, and 9.3% of all episodes required intensive care. These findings underscore the clinical and public health burden of DP, highlighting the need for tailored prevention and monitoring strategies across age groups.

The median age in our cohort is higher than that reported in several previous Spanish studies (Medina González et al., 2008; Caballero-Bermejo et al., 2022) and consistent with data from the SEMESTOX initiative showing an upward trend in the age of DP patients over the past 2 decades (Burillo Putze G et al., 2003). Female predominance is in line with earlier national cohorts, where DP episodes—especially those involving intentional self-poisoning—were more frequent in women, likely due to a combination of psychosocial, behavioral, and epidemiological factors (Supervía A et al., 2021; Couce Sánchez MJ et al., 2022).

Self-poisoning was the leading cause of DP in adults and in the pediatric population overall. In adolescents and young adults, this pattern may reflect increased psychological distress and reduced access to mental health services (Supervía A et al., 2021; Marín-Casino et al., 2024). Among adults over 60 years of age, accidental poisonings were more frequent (see Figure 1). This has been attributed to greater comorbidity and polypharmacy, which increase the risk of unintentional toxicity—especially in women, due to their longer life expectancy (Supervía A et al., 2021).Pediatric accidental exposures were frequently observed in children under 12 years of age, typically resulting from unsupervised access to household medications or dosing errors (Agarwal et al., 2020). These findings are consistent with earlier Spanish reports and underscore the importance of age-tailored prevention strategies (Supervía A et al., 2021; Aguilón-Leiva et al., 2022).

Across all categories of intentionality, benzodiazepines were the most frequently implicated drug class, followed by paracetamol derivatives and antipsychotics. These results align with previous national reports (Baeza et al., 2020; Couce Sánchez MJ et al., 2022). The frequent involvement of benzodiazepines may be explained by their widespread availability in households, ease of oral administration, and—particularly in older adults—the risk of accumulation associated with long half-life benzodiazepines, especially in the presence of renal or hepatic impairment (Juan Pablo Acuña, 2011; Carvajal and Müller-Ramírez, 2023).

Regarding paracetamol, it is widely used and available without a prescription in limited doses and package sizes. Its therapeutic margin is narrow, as the toxic dose is relatively close to the recommended therapeutic dose—particularly in pediatric patients, where small errors in dosing may lead to overdose (Casey et al., 2020). The presence of antipsychotics likely reflects underlying psychiatric morbidity in the affected population, as well as increased prescribing in both inpatient and outpatient mental health settings (Carvajal and Müller-Ramírez, 2023).

In adult self-poisoning episodes, benzodiazepines and antipsychotics were the predominant drug classes (Burillo Putze G et al., 2008; Medina González et al., 2008). These substances are frequently ingested in combination with other central nervous system depressants or analgesics to enhance the psychoactive or toxic effects, a behavior supported by evidence showing that co-users often seek potentiated intoxication—despite the well-known increased risk of sedation and respiratory depression (Jones et al., 2012; Gummin et al., 2017). These findings highlight the importance of prudent prescribing, early identification of individuals at risk of intentional polydrug overdose, and close monitoring. Risk-reduction strategies include limiting treatment duration, planning for withdrawal from the outset, and promoting non-pharmacological alternatives whenever appropriate (Vázquez Canales and Frutos Fernández, 2023; Choi et al., 2025).

In contrast, accidental poisoning episodes in adults were most commonly associated with opioids and cardiotonic glycosides, raising concerns about prescribing safety, polypharmacy, and treatment adherence in elderly patients. These episodes may arise from therapeutic mismanagement, drug–drug interactions, or age-related pharmacokinetic and pharmacodynamic changes (Medina González et al., 2008; Hernández-Calle et al., 2022; Mason et al., 2024).

About medications found in the pediatric population, benzodiazepines were the most frequently implicated drugs overall, however, paracetamol was the leading agent in intentional self-poisoning. These exposures are often related to medication access in the home environment, particularly to drugs prescribed to adult caregivers (Agarwal et al., 2020; Aguilón-Leiva et al., 2022).

Additionally, overdoses in children due to dosing device misinterpretation—particularly decimal point errors—have prompted regulatory responses. The Pharmacovigilance Risk Assessment Committee (PRAC) has recommended reinforcing caregiver instructions and updating package leaflets to prevent such incidents (EMA/PRAC/68905/2024). Beyond drugs, recent evidence highlights that other toxic agents, such as cannabis, are also an emerging concern in children, with toxicological, clinical, and medico-legal implications (Malta et al., 2025).

With respect to severity, most episodes in our study were clinically mild, with few cases requiring intensive care or with fatal endings. Similar ICU admission and mortality patterns have been described in previous studies in Spain (Banderas-Bravo et al., 2017; Socias Mir et al., 2021). However, this should not lead to underestimation of clinical severity. These patients could suffer altered consciousness and might require mechanical ventilation upon admission, particularly in the presence of significant comorbidities (Banderas-Bravo et al., 2017).

Concerning recurrent episodes, 12.5% of patients had more than one DP episode in our sample, mostly involving two separate admissions per patient. This recurrence rate is similar to previous national reports (Baeza et al., 2020). The most frequent causes of recurrence described in the literature are related to behavior around suicide attempts, and are often associated with underlying psychiatric disorders, emotional dysregulation, and psychosocial stressors (Finkelstein et al., 2016; Daly et al., 2020). These findings reinforce the need for targeted secondary prevention strategies in mental health (Kanehara et al., 2015).

Our trend analysis revealed an increase in DP admissions during 2020–2024, coinciding with the COVID-19 pandemic. The annual proportion of DP-related admissions rose from 1.11% in 2018–2019 to 1.97% in 2020–2024, with a statistically significant increase in both adult and pediatric populations. These findings are consistent with previous reports suggesting a shift in toxicological emergencies during the pandemic years (Puiguriguer Ferrando J et al., 2020; Caballero-Bermejo et al., 2022). Particularly concerning is the increase in adolescent self-poisoning episodes during lockdowns and the immediate post-pandemic period, a trend already described in the Spanish pediatric population (Fernández Lázaro JC et al., 2022). This phenomenon has been associated with increased social isolation, school difficulties, family stress, and reduced access to mental health services.

To our knowledge, this is the first study in Spain to analyze DP hospitalizations using the national hospital discharge registry (CMBD-AH), encompassing both adult and pediatric populations. Previous Spanish research has described trends in toxicological emergencies using emergency department data or clinical cohorts, but none have leveraged CMBD-AH to comprehensively examine inpatient burden, severity, recurrence, and post-pandemic trends. This hospital-based epidemiological approach offers valuable insights for toxicovigilance and healthcare planning.

### 4.1 Strengths and limitations

This single-center study provides a comprehensive overview of DP admissions over a 7-year period, capturing trends across the pre- and post-COVID-19 eras. Conducted at a large tertiary hospital with 1,146 inpatient beds and using the CMBD-AH—a validated hospital discharge registry—the study benefits from very comprehensive data and reliable diagnostic coding. The large sample size and extended timeframe allowed identification of relevant clinical patterns, including recurrence, ICU admissions, and mortality.

However, some limitations must be acknowledged. The retrospective design and reliance on administrative coding may have led to misclassification, particularly regarding the intentionality of poisoning. Episodes managed exclusively in outpatient settings or during brief emergency department visits not meeting admission criteria were not captured, potentially underestimating the overall burden. Nonetheless, the CMBD-AH classifies emergency department stays exceeding 16 h as hospital admissions, likely ensuring the inclusion of most clinically significant poisonings requiring observation or treatment.

Although findings may not be generalizable to other settings, the hospital’s status as a major referral center enhances the applicability of results to similar institutions. Despite these limitations, the study provides valuable data for toxicovigilance and supports evidence-based strategies in emergency and critical care.

## 5 Conclusion

This study provides a comprehensive overview of drug poisoning admissions at a Spanish tertiary hospital over a 7-year period, revealing relevant patterns by age, intentionality, and pharmacological groups. Although drug poisoning represents a small proportion of total hospitalization, they impose a considerable burden on healthcare services—particularly due to their recurrence nature and need for intensive care. These factors, along with the observed post-pandemic increase in admissions, underscore the importance of preventive strategies, mental health integration, and targeted interventions for high-risk populations.

Future research should validate these findings in multicenter settings, assess the role of polypharmacy and drug interactions, and combine CMBD-AH data with clinical and toxicological information to strengthen causal inferences. Integrating inpatient and outpatient toxicovigilance systems would enhance national monitoring capabilities.

## Data Availability

The raw data supporting the conclusions of this article will be made available by the authors, without undue reservation.
