# Supplementary material for: Hospital admissions for acute drug poisoning in adults and children: a 7-year retrospective analysis of hospital discharges at a tertiary center
Source: Front Toxicol. 2025 Oct 1;7:1672470. doi: 10.3389/ftox.2025.1672470 (PMC12521231; doi:10.3389/ftox.2025.1672470)
Supplement: Supplementary file 1 [file DataSheet1.pdf]

Supplementary Material

1. Supplementary Tables

Table 1: Classification of Drug Poisoning by Type used in the Study

Codes	Description	Codes	Description
T360	Penicillins	T4363	Methylphenidate
T361	Cephalosporins and other beta-lactam antibiotics	T4369	Other psychostimulants
T364	Tetracyclines	T438	Other psychotropic drugs
T365	Aminoglycosides	T4392	Unspecified psychotropic drug
T366	Rifampicins	T443	Other parasympatholytics and antispasmodics
T367	Antifungal antibiotics	T444	Adrenergic receptor alpha agonists
T368	Other systemic antibiotics	T446	Adrenergic receptor alpha antagonists
T3691	Unspecified systemic antibiotic	T447	Adrenergic receptor beta antagonists
T370	Sulfonamides	T448	Centrally acting and antiadrenergic agents
T371	Antimycobacterials	T4499	Other drugs acting on the autonomic nervous system
T373	Other antiprotozoal agents	T450	Antiallergics and antiemetics
T375	Antivirals	T451	Antineoplastics and immunosuppressants
T378	Other systemic anti-infectives and antiparasitics	T452	Vitamins
T380	Glucocorticoids and synthetic analogs	T454	Iron and iron compounds
T381	Thyroid hormones and substitutes	T4551	Anticoagulants
T383	Insulin and oral hypoglycemic agents	T4552	Antithrombotic drugs
T3889	Other synthetic hormones and substitutes	T458	Other systemically and hematologically acting agents
T3901	Acetylsalicylic acid	T460	Cardiotonic glycosides and similar acting drugs
T391	Paracetamol (acetaminophen)	T461	Calcium channel blockers
T392	Pyrazolone derivatives	T462	Other antiarrhythmic drugs
T3931	Propionic acid derivatives	T463	Coronary vasodilators
T3939	Other nonsteroidal anti-inflammatory drugs	T464	Angiotensin-converting enzyme inhibitors
T394	Antirheumatic drugs, not classified elsewhere	T465	Other antihypertensive drugs
T398	Other non-opioid analgesics and antipyretics	T466	Antilipemic and antiarteriosclerotic drugs
T399	Unspecified non-opioid analgesics and antipyretics	T4699	Other drugs mainly affecting the cardiovascular system
T400	Opium	T471	Other antacids and gastric secretion inhibitors
T402	Other opioids	T473	Saline and osmotic laxatives
T403	Methadone	T474	Other laxatives
T404	Other synthetic narcotics	T481	Skeletal muscle relaxants (neuromuscular blockers)
T406	Other and unspecified narcotics	T483	Antitussives
T411	Intravenous anesthetics	T484	Expectorants
T4129	Other general anesthetics	T486	Antiasthmatics
T413	Local anesthetics	T4899	Other drugs mainly acting on the respiratory system
T421	Iminostilbenes	T490	Antifungal, anti-infective, and anti-inflammatory local agents
T422	Succinimides and oxazolidinediones	T492	Astringents and local detergents
T423	Barbiturates	T495	Ophthalmologic drugs and preparations
T424	Benzodiazepines	T500	Mineralocorticoids and mineralocorticoid antagonists
T426	Other antiepileptic and hypnotic-sedative drugs	T501	Loop diuretics

T4274	Unspecified antiepileptic and hypnotic-sedative drugs	T502	Carbonic anhydrase inhibitors, benzothiazides, and other diuretics
T428	Antiparkinsonian drugs and other centrally acting muscle relaxants	T503	Electrolyte and caloric balance agents
T4301	Tricyclic antidepressants	T504	Drugs affecting uric acid metabolism
T4302	Tetracyclic antidepressants	T506	Antidotes and chelating agents
T4320	Unspecified antidepressants	T507	Analeptics and opioid receptor antagonists
T4321	Selective serotonin and norepinephrine reuptake inhibitors	T508	Diagnostic agents
T4322	Selective serotonin reuptake inhibitors	T5090	Unspecified drugs, medicines, and biological products
T4329	Other antidepressants	T5091	Multiple drugs, medicines, and biological products
T433	Phenothiazine-derived antipsychotics and neuroleptics	T5099	Other drugs, medicines, and biological products
T4350	Unspecified antipsychotics and neuroleptics	T50Z1	Immunoglobulin
T4359	Other antipsychotics and neuroleptics		
**The codes T36-50, if they end in:**<br>**1**: Accidental poisoning<br>**2**: Self poisoning<br>**4**: Undetermined poisoning			

Table 2. Hospital departments that discharged by age group and total

Department	<18 years, N (%)	≥ 18 years, N (%)	Total, N (%)
**Psychiatry**	32 (8.1)	1005 (3887)	1037 (34.6)
**Adult emergencies**	62 (15.7)	788 (30.4)	850 (28.4)
**Internal medicine**	3 (0.8)	229 (8.9)	232 (7.7)
**Adult oncology**	-	162 (6.2)	162 (5.4)
**Pediatric emergencies**	142 (35.9)	-	142 (4.7)
**Pediatrics**	84 (21.3)	3 (0.1)	87 (2.9)
**Adult ICU**	1 (0.3)	68 (2.6)	69 (2.3)
**Adult pulmonology**	1 (0.3)	63 (2.4)	64 (2.1)
**Adult cardiology**	-	53 (2.0)	53 (1.8)
**Pediatric psychiatry**	45 (11.4)	-	45 (1.6)
**Adult hematology**	-	41 (1.6)	41 (1.4)
**Adult nephrology**	-	29 (1.1)	29 (1.0)
**Adult traumatology**	-	24 (0.9)	24 (0.8)
**Gastroenterology**	-	21 (0.8)	21 (0.7)
**Adult neurology**	-	19 (0.7)	19 (0.6)
**Pediatric surgery**	14 (3.5)	-	14 (0.5)
**Thoracic surgery**	-	13 (0.5)	13 (0.4)
**General surgery**	-	11 (0.4)	11 (0.4)
**Vascular surgery**	-	11 (0.4)	11 (0.4)
**Pediatric ICU**	11 (2.8)	-	11 (0.4)
**Geriatrics**	-	11 (0.4)	11 (0.4)
**Neurosurgery**	-	8 (0.3)	8 (0.3)
**Urology**	-	7 (0.3)	7 (0.2)
**Gynecology and obstetrics**	-	7 (0.3)	7 (0.2)
**Cardiac surgery**	-	5 (0.2)	5 (0.2)
**Burn unit**	-	4 (0.2)	4 (0.1)
**Physical medicine and rehabilitation**	-	3 (0.1)	3 (0.1)
**Endocrinology**		2 (0.1)	2 (0.1)
**Plastic and reconstructive surgery**		2 (0.1)	2 (0.1)
**Home hospitalization**		2 (0.1)	2 (0.1)
**Rheumatology**		2 (0.1)	2 (0.1)
**Ophthalmology**		1 (0.0)	1 (0.0)
**Total episodes**	395 (100)	2594(100)	2989 (100)

Table 3. Admission episodes by age group and year of admission

		2018	2019	2020	2021	2022	2023	2024
**< 18 years**								
	Self-poisoning	15	9	23	34	36	51	46
	Accidental	25	22	11	18	24	23	11
	Undetermined	7	3	3	14	9	8	3
	**Total**	**47**	**34**	**37**	**66**	**69**	**82**	**60**
**≥ 18 years**								
	Self-poisoning	127	152	190	206	224	217	243
	Accidental	84	121	123	150	128	116	123
	Undetermined	55	41	48	69	74	56	47
	**Total**	**266**	**314**	**361**	**428**	**426**	**389**	**413**
**Total**								
	Self-poisoning	142	161	213	240	260	268	289
	Accidental	109	143	134	168	152	139	134
	Undetermined	62	44	51	83	83	64	50
	**Total**	**313**	**348**	**398**	**491**	**495**	**471**	**473**

Table 4. Most common medical and pathological histories coded during the study

	<18 years N		≥ 18 years N		Total N
**Anxiety disorder**	53	**Nicotine dependence**	733	**Nicotine dependence**	751
**Major depressive disorder**	39	**Anxiety disorder**	524	**Essential hypertension**	531
**Personal history of self-harm**	37	**Essential hypertension**	523	**Anxiety disorder**	594
**Suicidal ideation**	29	**Dyslipemia**	522	**Dyslipemia**	522
**Adjustment disorder**	27	**Type 2 diabetes mellitus**	436	**Major depressive disorder**	445
**Attention deficit disorder**	25	**Major depressive disorder**	541	**Type 2 diabetes mellitus**	422
**Eating disorder**	25	**Alcohol abuse**	366	**Alcohol abuse**	377
**Cannabis abuse**	21	**Personal history of self-harm**	292	**Personal history of self-harm**	329
**Acute renal failure**	15	**Acute renal failure**	270	**Acute renal failure**	285
**Autistic disorder**	10	**Heart failure**	224	**Suicidal ideation**	216
**Borderline personality disorder**	8	**Borderline personality disorder**	201	**Cannabis abuse**	209

Table 5: Drugs associated with fatal episodes by intentionality and age group.

Intentionality	Drug 1	Drug 2	Drug 3	Pediatric n (%)	Adult n (%)	Total n (%)
Accidental	Opioids	-	-	0 (0.0)	17 (19.8)	17 (19.3)
Accidental	Cardiotonic glycosides	-	-	0 (0.0)	8 (9.3)	8 (9.2)
Accidental	Antineoplastics and Immunosuppressants	-	-	1 (50.0)	4 (4.6)	5 (5.7)
Accidental	Insulin and Hypoglycemics	-	-	0 (0.0)	5 (5.7)	5 (5.7)
Accidental	Benzodiazepines	-	-	0 (0.0)	4 (4.6)	4 (4.6)
Undetermined	Medications and Biological Products	-	-	0 (0.0)	4 (4.6)	4 (4.6)
Accidental	Anticoagulants	-	-	0 (0.0)	4 (4.6)	4 (4.6)
Undetermined	Opioids	-	-	0 (0.0)	4 (4.6)	4 (4.6)
Accidental	Narcotics	-	-	0 (0.0)	4 (4.6)	4 (4.6)
Undetermined	Benzodiazepines	-	-	0 (0.0)	3 (3.5)	3 (3.4)
Undetermined	Antineoplastics and Immunosuppressants	-	-	0 (0.0)	3 (3.5)	3 (3.4)
Self-poisoning	Insulin and Hypoglycemics	-	-	0 (0.0)	3 (3.5)	3 (3.4)
Accidental	Antipsychotics and Neuroleptics	-	-	0 (0.0)	3 (3.5)	3 (3.4)
Accidental	Medications and Biological Products	-	-	1 (50.0)	2 (2.3)	3 (3.4)
Self-poisoning	Benzodiazepines	-	-	0 (0.0)	2 (2.3)	2 (2.3)
Accidental	β-adrenoceptor antagonists	-	-	0 (0.0)	2 (2.3)	2 (2.3)
Accidental	Systemic antibiotics	-	-	0 (0.0)	2 (2.3)	2 (2.3)
Accidental	Selective Serotonin Reuptake Inhibitors	-	-	0 (0.0)	1 (1.2)	1 (1.1)
Undetermined	Paracetamol	-	-	0 (0.0)	1 (1.2)	1 (1.1)
Accidental	Antiarrhythmics	-	-	0 (0.0)	1 (1.2)	1 (1.1)
Undetermined	Antifungals	-	-	0 (0.0)	1 (1.2)	1 (1.1)
Accidental	Antiepileptics and Hypnotics	-	-	0 (0.0)	1 (1.2)	1 (1.1)
Accidental	Analeptics and Opioids Antagonist	-	-	0 (0.0)	1 (1.2)	1 (1.1)
Undetermined	Narcotics	-	-	0 (0.0)	1 (1.2)	1 (1.1)
Undetermined	Medications and Biological Products	-	-	0 (0.0)	1 (1.2)	1 (1.1)
Accidental	Antimycobacterials	-	-	0 (0.0)	1 (1.2)	1 (1.1)
Self-poisoning	Benzodiazepines	Narcotics	-	0 (0.0)	1 (1.2)	1 (1.1)
Self-poisoning	Analgesic and Antipyretic	Antipsychotics and Neuroleptics	Medications and Biological Products	0 (0.0)	1 (1.2)	1 (1.1)
Accidental	Opioids	Benzodiazepines	-	0 (0.0)	1 (1.2)	1 (1.1)
**Total episodes**				2 (100)	86 (100)	88 (100)

Note: The presence of a drug poisoning diagnosis does not necessarily imply a causal relationship with death or ICU admission

Table 6: Most frequent drug combinations

Intentionality	Drug 1	Drug 2	Drug 3	N (%)
**Self-poisoning**	Non-Steroidal Anti-inflammatory Drugs	Benzodiazepines		15
**Self-poisoning**	Benzodiazepines	Medications and biological products		6
**Self-poisoning**	Paracetamol	Propionic Acid Derivatives		5
**Self-poisoning**	Antiepileptics and Hypnotics	Benzodiazepines		5
**Self-poisoning**	Paracetamol	Benzodiazepines		5
**Self-poisoning**	Antipsychotics and Neuroleptics	Benzodiazepines		4
**Self-poisoning**	Antidepressants	Benzodiazepines		4
**Self-poisoning**	Antipsychotics and Neuroleptics	Medications and biological products		4
**Undetermined**	Antidepressants	Methadone		
**Self-poisoning**	Paracetamol	Medications and biological products		3
**Self-poisoning**	Antidepressants	Antipsychotics and Neuroleptics		3
**Self-poisoning**	Antipsychotics and Neuroleptics	Angiotensin-Converting Enzyme Inhibitor	Selective Serotonin Reuptake Inhibitors	3
**Self-poisoning**	Benzodiazepines	Narcotics		3
**Self-poisoning**	Benzodiazepines	Opiods		3
**Others**				215

SUMMARY OF INTENTIONALITY
Self-inflicted: 251 (78.7%)
Accidental: 38 (11.9%)
Undetermined: 30(9.4%)
Total: 319 (100%)
